# Light action spectrum on oxidative stress and mitochondrial damage in A2E-loaded retinal pigment epithelium cells

**DOI:** 10.1038/s41419-018-0331-5

**Published:** 2018-02-19

**Authors:** Mélanie Marie, Karine Bigot, Claire Angebault, Coralie Barrau, Pauline Gondouin, Delphine Pagan, Stéphane Fouquet, Thierry Villette, José-Alain Sahel, Guy Lenaers, Serge Picaud

**Affiliations:** 1 Sorbonne Université, INSERM, CNRS, Institut de la Vision, 17 rue Moreau, 75012 Paris, France; 2 INSERM U1051 - Institut des Neurosciences de Montpellier, 34091 Montpellier, France; 3 Essilor International R&D, 94220 Charenton-Le-Pont, France; 4 CHNO des Quinze-Vingts, DHU Sight Restore, INSERM-DGOS 1423, 75012 Paris, France; 5 Fondation Ophtalmologique Rothschild, 75019 Paris, France; 6 Department of Ophthalmology, The University of Pittsburgh School of Medicine, Pittsburgh, PA 15213 USA; 7 Equipe MitoLab, Pôle de Recherche et d’Enseignement en Médecine Mitochondriale, Institut MitoVasc, Université d’Angers, UMR CNRS 6015, INSERM U1083, 49933 Angers, France

## Abstract

**Aims:**

Blue light is an identified risk factor for age-related macular degeneration (AMD). We investigated oxidative stress markers and mitochondrial changes in A2E-loaded retinal pigment epithelium cells under the blue–green part of the solar spectrum that reaches the retina to better understand the mechanisms underlying light-elicited toxicity.

**Results:**

Primary retinal pigment epithelium cells were loaded with a retinal photosensitizer, AE2, to mimic aging. Using a custom-made illumination device that delivers 10 nm-wide light bands, we demonstrated that A2E-loaded RPE cells generated high levels of both hydrogen peroxide (H_2_O_2_) and superoxide anion (O_2_^•−^) when exposed to blue–violet light. In addition, they exhibited perinuclear clustering of mitochondria with a decrease of both their mitochondrial membrane potential and their respiratory activities. The increase of oxidative stress resulted in increased levels of the oxidized form of glutathione and decreased superoxide dismutase (SOD) and catalase activities. Furthermore, mRNA expression levels of the main antioxidant enzymes (SOD2, catalase, and GPX1) also decreased.

**Conclusions:**

Using an innovative illumination device, we measured the precise action spectrum of the oxidative stress mechanisms on A2E-loaded retinal pigment epithelium cells. We defined 415–455 nm blue–violet light, within the solar spectrum reaching the retina, to be the spectral band that generates the highest amount of reactive oxygen species and produces the highest level of mitochondrial dysfunction, explaining its toxic effect. This study further highlights the need to filter these wavelengths from the eyes of AMD patients.

## Introduction

Age-related macular degeneration (AMD) is a major cause of blindness in elderly people^1,2^. Light is now widely considered to be a risk factor for this multifactorial disease in addition to age, genetics, smoking, and diet^3^. Early stages of AMD are characterized by the accumulation of yellow fluorescent deposits in the macula. These deposits contain lipofuscin, a residue that accumulates with age in retinal pigment epithelium (RPE) cells as a consequence of the incomplete digestion of photoreceptor outer segments^4^. Its intracellular accumulation enhances cellular sensitivity to light radiation^5^, providing a possible cellular mechanism to explain the RPE dysfunction that causes AMD^2^. This cellular photosensitization is partly attributed to A2E, a prominent retinoid constituent of lipofuscin^6–9^, which displays absorbance peaks at 335 and 435 nm^10^. The consecutive production of reactive oxygen species (ROS) by A2E photosensitization was demonstrated in pure preparation of lipofuscin granules and in synthesized A2E^7,11^ or even in RPE cells^8,12^. When RPE cells are incubated in the presence of A2E, green autofluorescent vesicles appear in the cell body under blue light indicative of A2E uptake into lysosomes^13^. This A2E uptake is dose dependent and does not saturate up to 40 µM in the incubation medium^13^.”

Within the light spectrum, the blue range has been defined in several epidemiological studies as a risk factor in AMD^3,14–18^ in agreement with the blue-light sensitivity of A2E leading to ROS accumulation and cell death^8,10,19–25^. These recent results suggested that blue-light filters could limit the risk of AMD or its dramatic progression^26,27^. However, blue light is also important for vision, especially in mesopic or scotopic conditions and for the regulation of circadian rhythms, questioning therefore the use of such broadband deep-tinted blue-cut filters^27^.

To further precise toxic wavelengths within the blue range, we recently developed a light-emitting device to apply 10 nm light bands on cell cultures^13^. A2E-loaded primary RPE cells were thus exposed to 10 nm-wide bands of light that were normalized to the corresponding daylight reaching the retina, taking into account the natural filtering of the eye media. In this study, we thus showed that the loss-of-viability and induction of apoptosis were highest in the narrow spectral range from 415 to 455 nm.

To verify these results on other molecular and cellular parameters and to identify biomarkers to assess filter-expected cell protection, we measured several markers of oxidative stress in A2E-loaded RPE cells and generated for some their light spectrum of induction.

## Results

### High levels of intracellular ROS after blue–violet light exposure

**Fig. 1:**
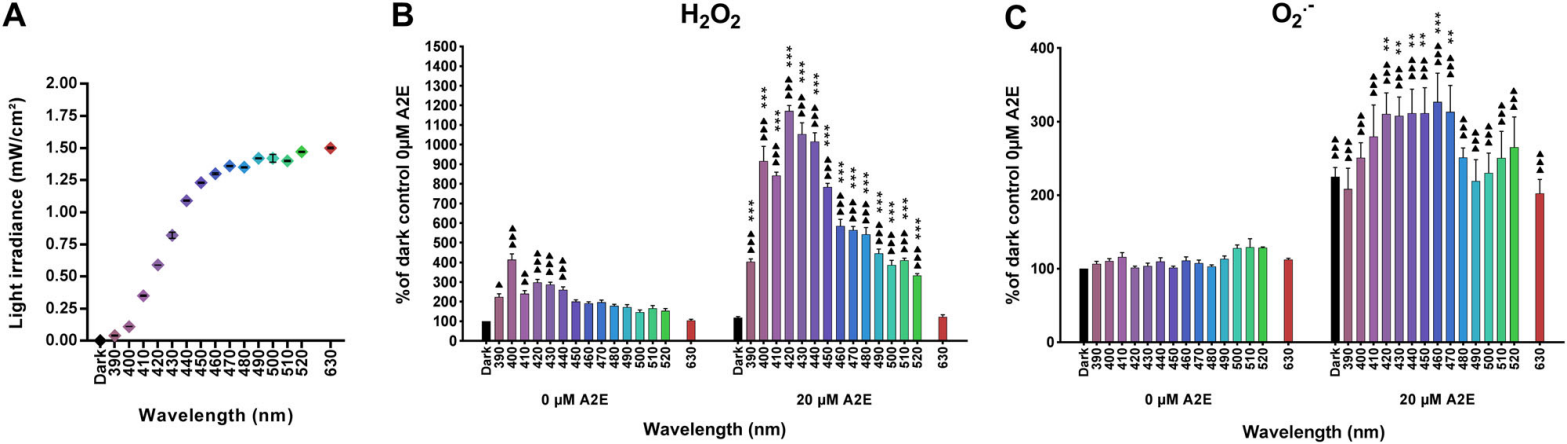
ROS levels in light-exposed RPE cells. Hydrogen peroxide (H_2_O_2_, **b**, *n* = 3–5) and superoxide anion (O_2_^•^^−^, **c**, *n* = 4–5) levels were measured in RPE cells loaded with 20 µM A2E and exposed to light (**a**, *n* = 16) for 15 h. Differences between samples and dark controls were considered to be significant when *p* < 0.05 (▲/*), *p* < 0.01 (▲▲/**), or *p* < 0.001 (▲▲▲/***). Triangles (▲) refer to the difference with the untreated dark control and stars (*) to A2E-treated dark control. Each 10 nm spectral band is designated by its central wavelength

To further assess the spectral dependency of phototoxicity in A2E-loaded RPE cells, we first measured the level of two major ROS: hydrogen peroxide (H_2_O_2_) and superoxide anion (O_2_^•−^). In these experiments, visible light exposure was reduced from 18 to 15 h to limit cell death (Fig. 1a). In the absence of A2E, light-induced low levels of H_2_O_2_ in RPE cells throughout the tested range of 390–520 nm, with a fourfold maximum at 400 nm (Fig. 1b). The differences were statistically significant except between 450 and 630 nm. In A2E-loaded RPE cells, light exposure generated much more H_2_O_2_ reaching levels that were up to 10-fold higher than the control level in the dark. The greatest increases occurred for the 10 nm bands centered at 420, 430, and 440 nm, with a peak at 420 nm. We observed no difference at 630 nm. A2E therefore greatly increased H_2_O_2_ production, shifting the peak sensitivity toward the peak of A2E photosensitization.

Surprisingly, light did not induce O_2_^•−^ production in the absence of A2E (Fig. 1c). A2E (20 µM) loaded cells had a twofold higher level of O_2_^•−^ than those without A2E, even when maintained in the dark. Following light exposure, O_2_^•−^ production further increased, with a plateau for bands of wavelengths centered from 420 to 470 nm. In contrast to the 10-fold increase in H_2_O_2_ levels, the increase in O_2_^•−^ levels was very modest (below 1.5-fold). These results demonstrate that the presence of A2E greatly increased light-induced ROS production, especially H_2_O_2_. They further show that ROS production was maximal in the blue–violet light range.

### Impact of light on mitochondria

**Fig. 2:**
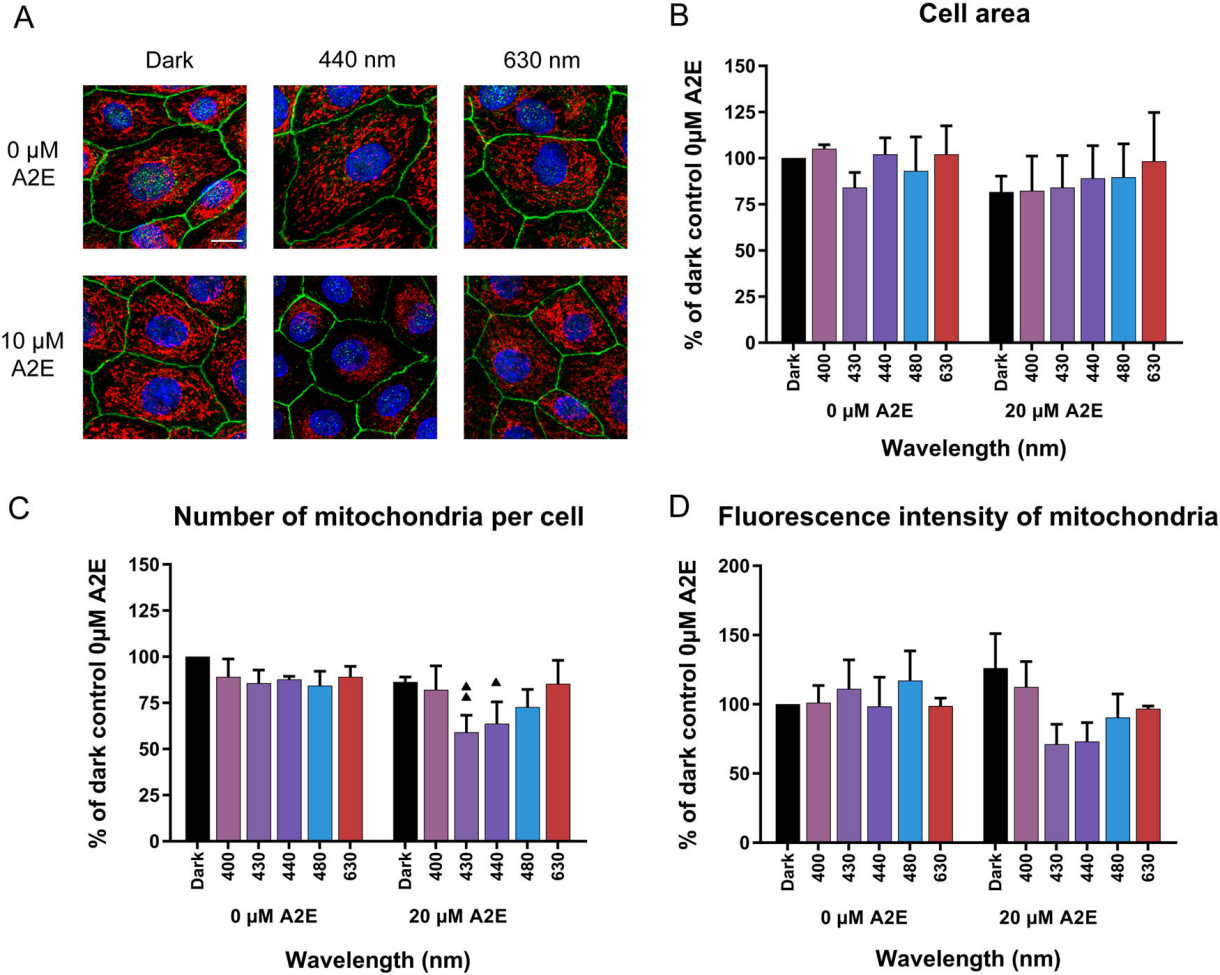
Impact of light on mitochondria in A2E-loaded RPE cells. **a** Confocal images of RPE cells exposed to A2E and light (440 nm and 630 nm) showing changes in mitochondrial distribution at 440 nm. Mitochondria were immunostained with an anti-ATP synthase antibody, tight junctions with an anti-ZO-1 antibody, nuclei were counter-stained with DAPI and images were acquired using confocal microscopy. Scale bar represents 10 µm. Quantification of the cell area **b**, the number of mitochondria per cell **c** and the cell mitochondrial fluorescence intensity **d** using the ‘Cell’ module of Imaris software. *n* = 3. Differences between samples and dark controls were considered to be significant when *p* < 0.05 (▲/*), *p* < 0.01 (▲▲/**), or *p* < 0.001 (▲▲▲/***). Triangles (▲) refer to the difference with the untreated dark control and stars (*) to A2E-treated dark control. Each 10 nm spectral band is designated by its central wavelength

In A2E-loaded RPE cells, light-induced a decrease in the cellular ATP content at 430 nm but not at other tested wavelengths (Supplementary Information 1). To further understand the underlying mechanism of this ATP reduction, we examined mitochondria starting with their distribution upon light exposure to blue–violet light, such as 430 and 440 nm (Fig. 2a). Under dark control conditions, mitochondria were homogeneously distributed throughout the cytoplasm with a classic tubular shape. In the absence of A2E, light exposure did not affect the cellular distribution of mitochondria. This homogenous distribution was not affected by the addition of A2E (10 µM) in darkness or after light exposure at 630 nm. However, A2E-loaded cells showed a striking constriction of the mitochondrial network within a perinuclear area upon light exposure at 440 nm (Fig. 2a). As shown by the cell area quantification (Fig. 2b) the constriction of the mitochondrial network was not due to a decrease of cell area as this parameter remained stable among all the tested conditions. However, the automatic quantification of the number of mitochondria per cell indicated that A2E-loaded cells exposed to blue light (430 and 440 nm) exhibited a significantly reduced number of mitochondria (Fig. 2c). The perinuclear migration of mitochondria provides the first evidence of the light-induced alteration of mitochondria in A2E-loaded cells exposed to blue light. In view of these small, but statistically significant differences, we did not extend this quantification to the full spectrum, but restricted it to the main wavelengths of the phototoxic action spectrum.

### Mitochondrial respiration

**Fig. 3:**
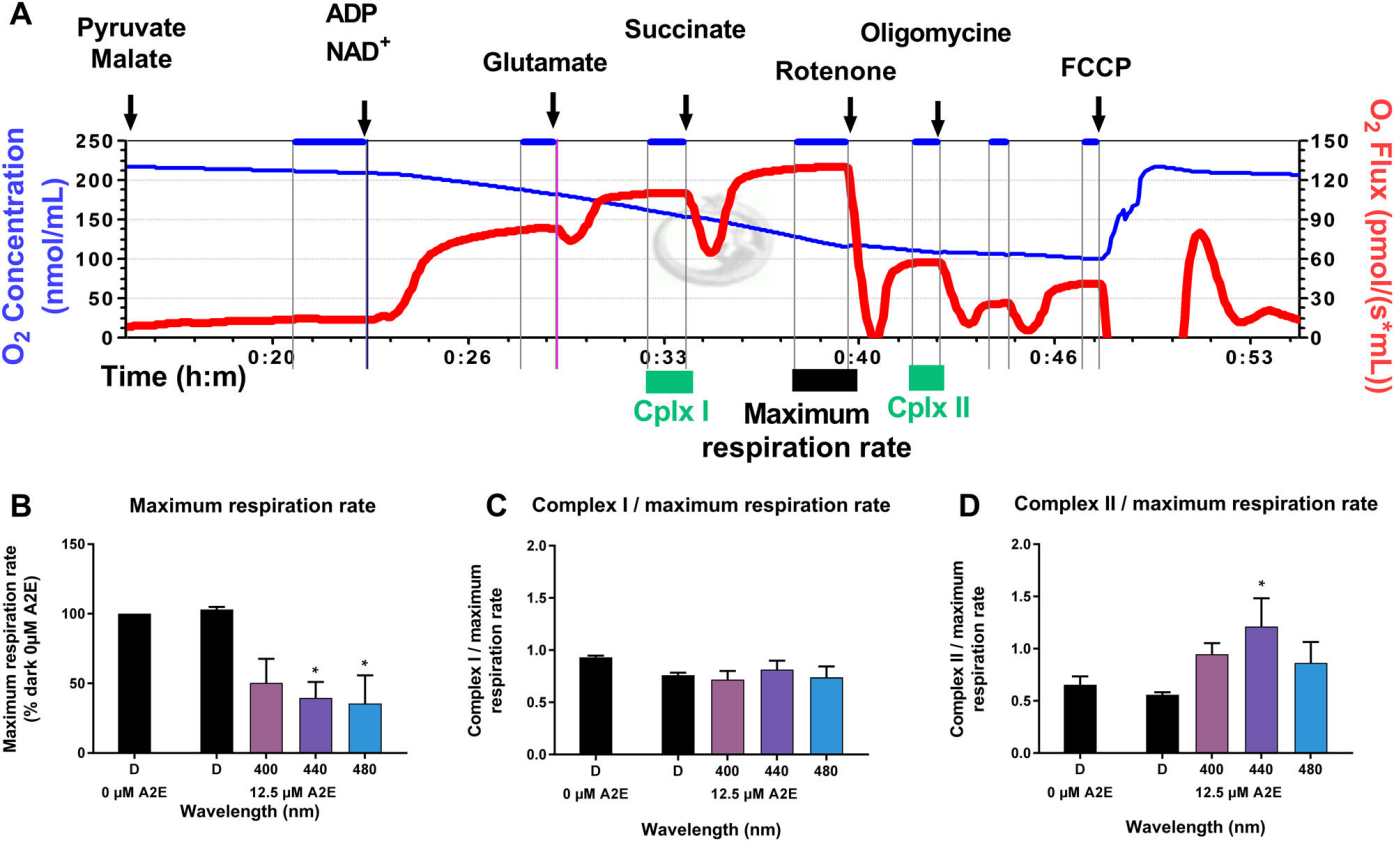
Mitochondrial respiration in light-exposed RPE cells. Representative results of mitochondrial oxygen consumption measured with an oxygraph: the oxygen concentration in a closed chamber is represented by the blue line and oxygen flux by the red line **a**. Maximum respiration rate (**b**) and activities of mitochondrial Complex I and II **c**, **d** were determined after the addition of various exogenous substrates and drugs immediately after light exposure. *n* = 4–5. Differences between samples and dark controls were considered to be significant when *p* < 0.05 (▲/*), *p* < 0.01 (▲▲/**), or *p* < 0.001 (▲▲▲/***). Triangles (▲) refer to the difference with the untreated dark control and stars (*) to A2E-treated dark control. Each 10 nm spectral band is designated by its central wavelength

We assessed mitochondrial function by measuring oxidative phosphorylation in A2E-loaded RPE cells after 15 h of light exposure using an oxygraph. A lower concentration of A2E (12.5 µM) was used instead of 20 µM to induce a more moderate light toxicity and thus facilitate the measurements. The maximal respiration rate was measured by quantifying oxygen consumption after successively applying malate, pyruvate, ADP, NAD^+^, glutamate, and succinate to permeabilized RPE cells (Fig. 3a). The measurements were first taken on RPE cells maintained in darkness without A2E and subsequent measurements were normalized to this initial measurement. The respiration rate was not affected in A2E-loaded RPE cells maintained in darkness (Fig. 3b). In contrast, the rate was markedly lower in A2E-loaded RPE cells exposed to blue light than those maintained in darkness and the differences were statistically significant at 440 and 480 nm (Fig. 3b). We added rotenone to block Complex I of oxidative phosphorylation to more precisely define this effect on oxidative phosphorylation (Fig. 3a). The decreases were normalized to the total respiration rate. Surprisingly, these ratios were not modified under any conditions tested, including A2E-loaded cells exposed to blue light (Fig. 3c) suggesting that Complex I activity was suppressed by blue light to the same extent as the total respiration rate. These measurements provide evidence that blue–violet light affect oxidative phosphorylation through Complex I in A2E-loaded cells. We measured the activity of Complex II in the oxygraph chamber after applying rotenone and then normalized these values to the total respiration rate (Fig. 3a). The ratio was significantly higher in A2E-loaded cells exposed to blue light at 440 nm (Fig. 3d). This increase indicates that Complex II was less affected by blue light than the total respiration rate or Complex I. Although the experiments were limited to a few wavelengths due to the complexity of the measurements, our results show that the effect of light on Complex I and II of the mitochondrial electron-transport chain could both contribute to, and result from, ROS production in A2E-loaded RPE cells exposed to blue light.

### Mitochondrial membrane potential

**Fig. 4:**
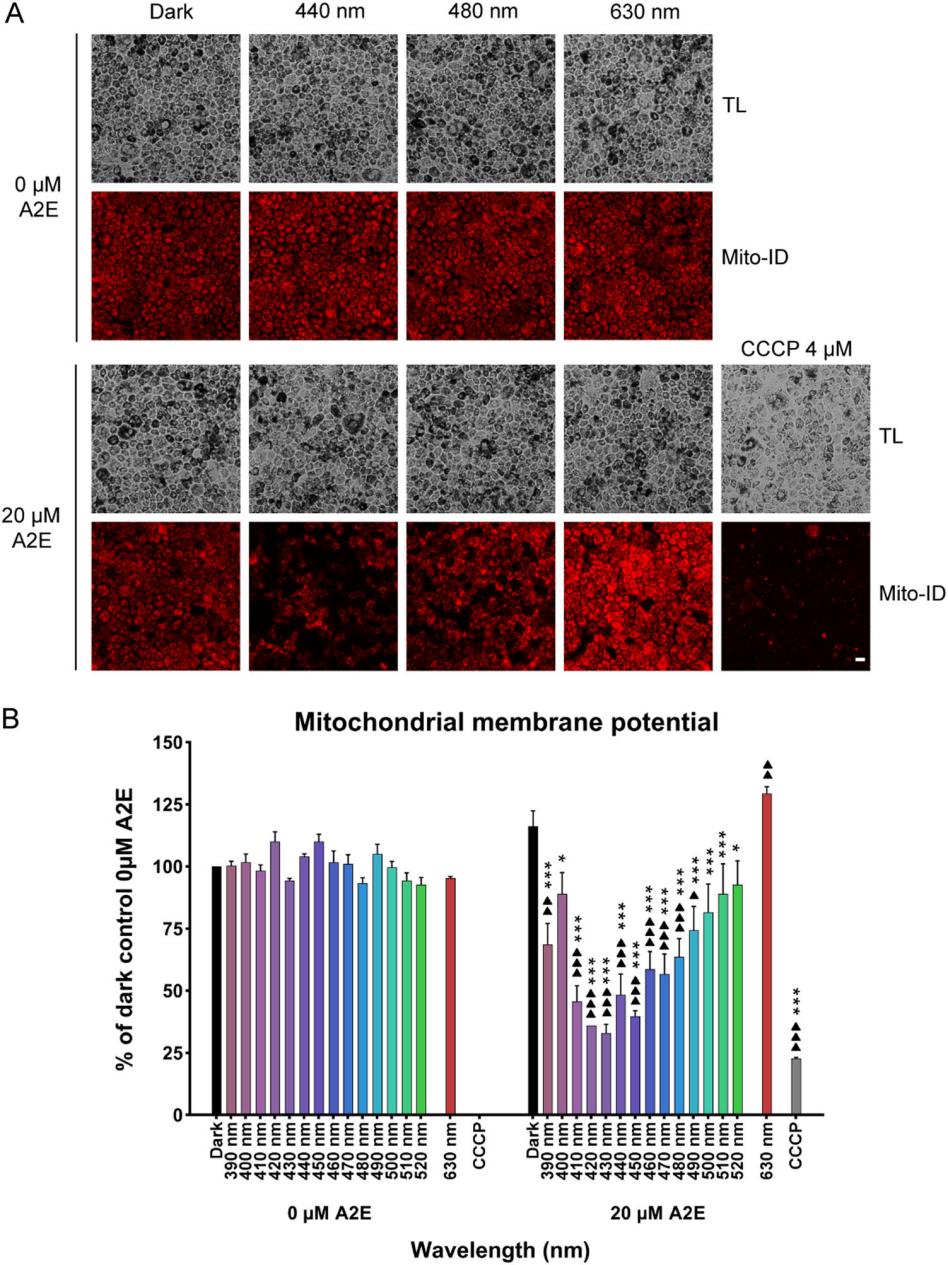
Mitochondrial membrane potential in light-exposed RPE cells. **a** The mitochondrial membrane potential was measured by quantifying dye aggregates on a microplate reader in RPE cells exposed to light for 15 h **b**. Scale bar represents 20 µm. TL: transmitted light. *n* = 3. Differences between samples and dark controls were considered to be significant when *p* < 0.05 (▲/*), *p* < 0.01 (▲▲/**), or *p* < 0.001 (▲▲▲/***). Triangles (▲) refer to the difference with the untreated dark control and stars (*) to A2E-treated dark control. Each 10 nm spectral band is designated by its central wavelength

Mitochondrial function relies highly on the mitochondrial membrane potential. We therefore investigated whether this important functional parameter of mitochondria was affected by light exposure by measuring the mitochondrial membrane potential with the Mito-ID dye. Light exposure did not modify the fluorescence of the dye in the absence of A2E incubation, irrespective of the spectral band (Fig. 4a, b). We performed a positive control to verify the quality of the Mito-ID dye by applying the mitochondrial uncoupler, CCCP. CCCP treatment resulted in markedly lower dye fluorescence in RPE-cells (Fig. 4a), which was confirmed by quantification (Fig. 4b). Surprisingly, the mitochondrial membrane potential in RPE cells was slightly higher following A2E incubation. There was a similar increase for A2E-loaded RPE cells exposed to red light (630 nm) (Fig. 4a, b). In contrast, Mito-ID fluorescence was significantly lower in A2E-loaded cells exposed to blue–violet light at 440 nm (Fig. 4a), which was confirmed by fluorescence quantification (Fig. 4b). Among all tested wavelengths, we observed a decrease in the membrane potential in the blue range with a plateau between 410 and 450 nm (Fig. 4b). These results indicate that mitochondrial function was altered in A2E-loaded cells exposed to blue–violet light with a maximum effect between 410 and 450 nm.

**Fig. 5:**
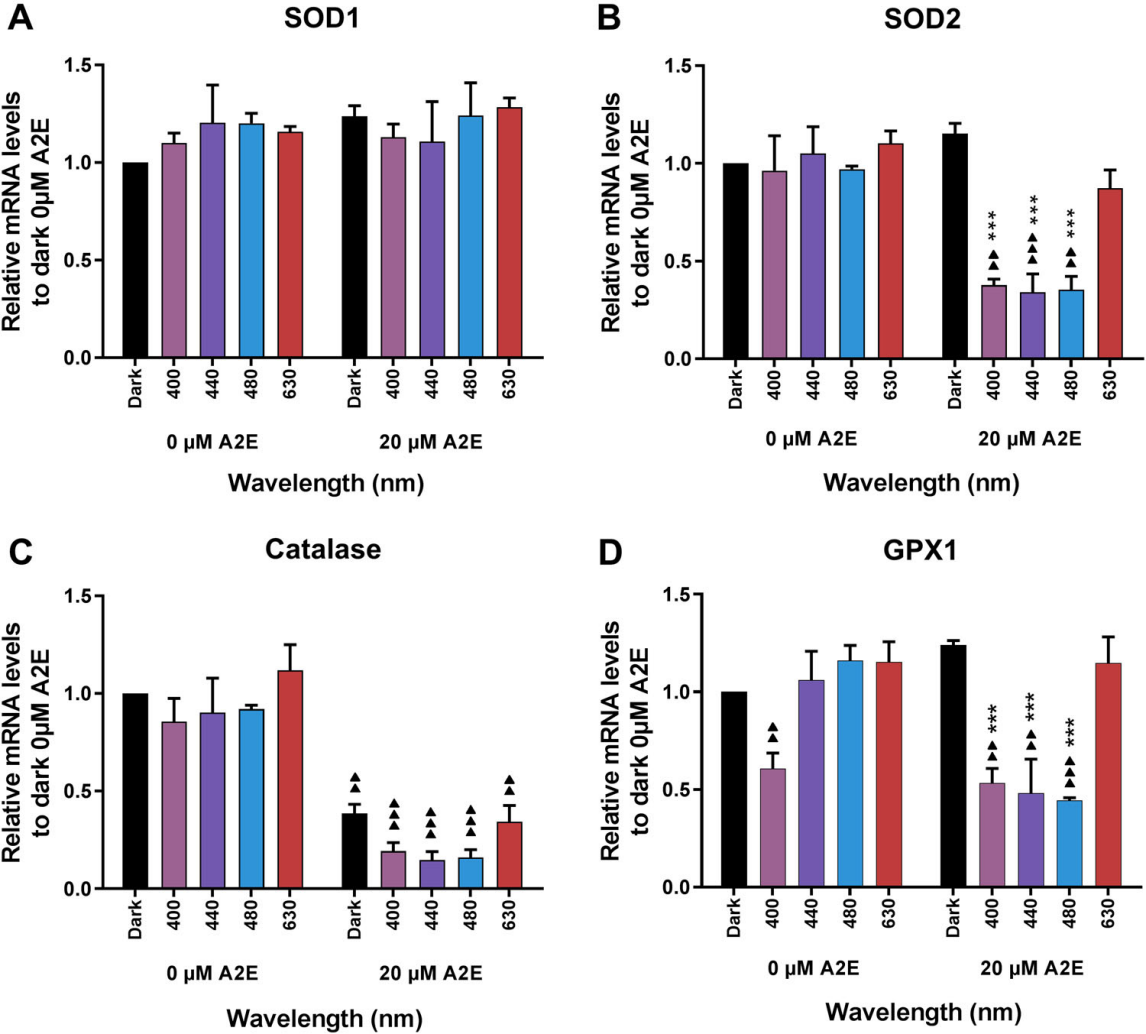
mRNA expression levels of oxidative stress defense proteins in light-exposed RPE cells. We measured the mRNA expression levels for SOD1 **a**, SOD2 **b**, catalase **c**, and GPX1 **d** proteins in RPE cells after 15 h of light exposure in the presence or absence of A2E (20 µM). All values were normalized to the expression level of ribosomal 18S RNA under the same experimental conditions and then normalized to the expression levels of RPE cells maintained in darkness without A2E. *n* = 3. Differences between samples and dark controls were considered to be significant when *p* < 0.05 (▲/*), *p* < 0.01 (▲▲/**), or *p* < 0.001 (▲▲▲/***). Triangles (▲) refer to the difference with the untreated dark control and stars (*) to A2E-treated dark control. Each 10 nm spectral band is designated by its central wavelength

### Antioxidant mechanisms

The antioxidant mechanisms include the catalysis of superoxide anions to hydrogen peroxide by SOD, the degradation of hydrogen peroxide by catalase, and the reduction of ROS by glutathione. Because gene expression has been reported to be affected by A2E^19^, we measured the mRNA levels for proteins involved in antioxidant mechanisms, including SOD1, SOD2, catalase, and glutathione peroxidase 1 (which reduces glutathione after its oxidation by ROS). In the absence of A2E, light did not induce any significant modification of mRNA levels of the four tested genes except GPX1, which was significantly lower following exposure at 400 nm (Fig. 5). In darkness, A2E treatment did not affect the mRNA levels of SOD1, SOD2, or GPX1, whereas the level of catalase mRNA was significantly lower than in non-A2E-treated controls. In contrast, the levels of SOD2, catalase, and GPX1 mRNA were markedly downregulated (at least twofold) in A2E-loaded cells exposed to 400, 440, and 480 nm. Catalase mRNA expression level after light exposure was decreased to a higher level than the reduction induced by A2E incubation alone. The level of SOD1 mRNA was not altered under any of the tested conditions. These effects may be attributed to the oxidative stress induced by blue–violet light since red light exposure (630 nm) did not modify the mRNA expression levels for any of the studied genes. Altogether, these results show that blue-light exposure affected oxidative defense mechanisms by reducing mRNA expression levels of the three main proteins (SOD2, catalase, and GPX1) involved in defensive mechanisms against oxidative stress.

**Fig. 6:**
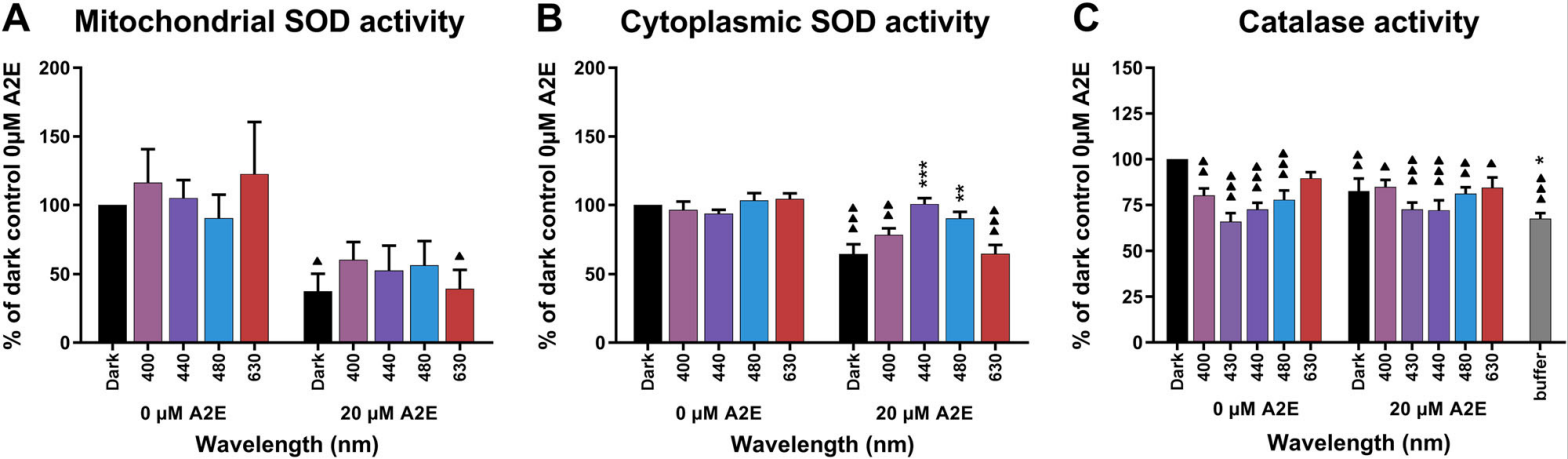
Catalase and superoxide dismutase (SOD) activities in light-exposed RPE cells. SOD (**a** and **b**, *n* = 5) and catalase (**c**, *n* = 5) enzymatic activities were measured in RPE cells after 15 h of light exposure in the presence or absence of A2E (20 µM). Differences between samples and dark controls were considered to be significant when *p* < 0.05 (▲/*), *p* < 0.01 (▲▲/**), or *p* < 0.001 (▲▲▲/***). Triangles (▲) refer to the difference with the untreated dark control and stars (*) to A2E-treated dark control. Each 10 nm spectral band is designated by its central wavelength

We also investigated the impact of light exposure on antioxidant defense mechanisms by measuring SOD and catalase enzymatic activity. The mitochondrial fraction of RPE cells contained mostly SOD2, whereas SOD1 was more abundant in the cytoplasmic fraction. SOD activity was lower in the mitochondrial (1–6 U/mL) than in the cytoplasmic fraction (8–21 U/mL, data not shown). In the absence of A2E treatment, light exposure (400, 440, 480, and 630 nm) did not modify the level of SOD activity in either the mitochondrial or cytoplasmic fractions (Fig. 6a, b). Surprisingly, A2E treatment significantly reduced mitochondrial SOD enzymatic activity of RPE cells maintained in darkness by threefold and cytoplasmic SOD activity by almost twofold. These differences were statistically significant. However, exposure of A2E-loaded RPE cells to blue light (400–480 nm) increased mitochondrial and cytoplasmic SOD activities, such that, under the 440 nm blue–violet light exposure, it returned back to control levels prior to the A2E incubation for the cytoplasmic fraction. This increase was not as great in the mitochondrial fraction, such that it did not reach control levels. Exposure to 630 nm light did not affect SOD activity, which remained at the same low level as in A2E-loaded cells maintained in darkness. These results suggest that A2E impaired mostly mitochondrial SOD activity, but that the oxidative stress generated by blue-light exposure on A2E-loaded cells may transiently stimulate SOD activity.

The results of catalase enzymatic activity in whole-cell protein extracts (Fig. 6c) were different from that of SOD. First, the values were very close to those of buffer alone, and thus the results must be interpreted with caution. Second, blue–violet light exposure further decreased its level, even in the absence of A2E pre-incubation. This effect was maximal at 430, and 440 nm in the blue–violet range. We observed a similar decrease of catalase activity in A2E-loaded RPE cells, in the same blue–violet range (430, 440 nm). Although these results need to be interpreted with caution, the effect on the catalase appears to be more highly related to the wavelength of exposure rather than the oxidative stress generated by A2E photosensitization. No significant change in protein levels of enzymes involved in antioxidant defense mechanisms was detected by western blotting immediately or 24 h after the end of light exposure (data not shown).

**Fig. 7:**
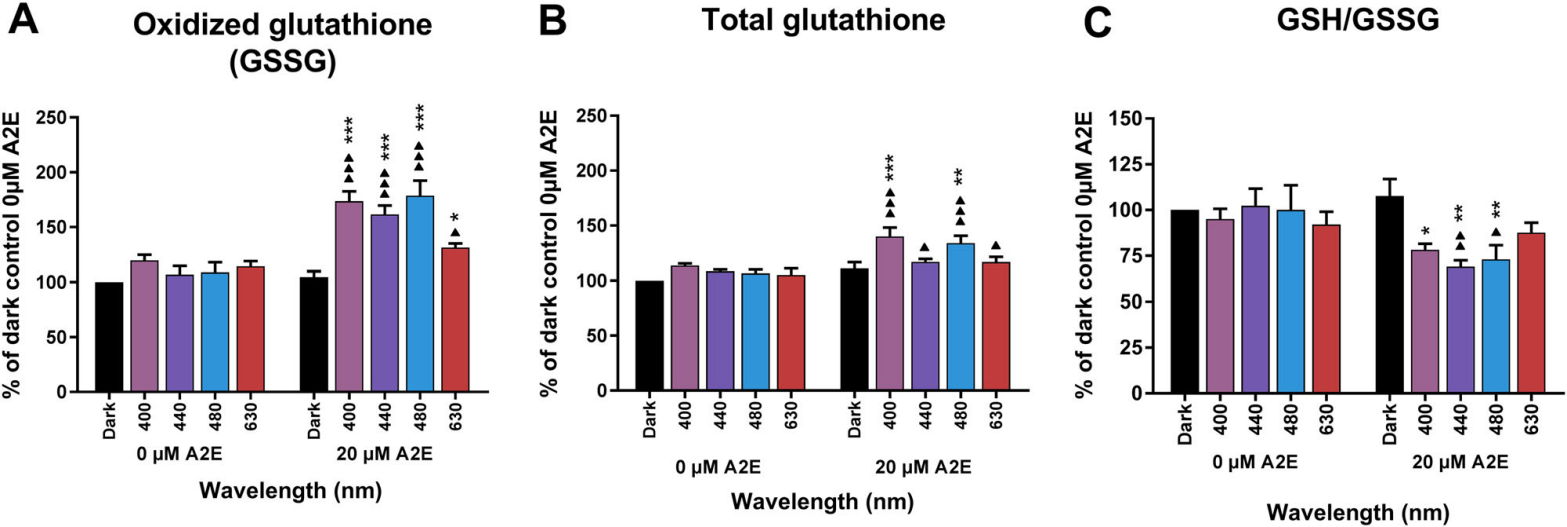
Total, oxidized, and reduced glutathione levels in light-exposed RPE cells. Oxidized (GSSG, **a**) and total glutathione levels **b** were measured in A2E-loaded cells after a 15-h light exposure. Glutathione ratios (GSH/GSSG, **c**) were calculated according to the manufacturer’s instructions. *n* = 3. Differences between samples and dark controls were considered to be significant when *p* < 0.05 (▲/*), *p* < 0.01 (▲▲/**), or *p* < 0.001 (▲▲▲/***). Triangles (▲) refer to the difference with the untreated dark control and stars (*) to A2E-treated dark control. Each 10 nm spectral band is designated by its central wavelength

The oxidized form of glutathione (GSSG) is a direct indicator of cellular oxidative stress, because glutathione is converted from the reduced state (GSH) to the oxidized form (GSSG) under oxidative stress. Light exposure of RPE cells, without A2E treatment, to the tested wavelengths (400, 440, 480, and 630 nm) did not affect GSSG content (Fig. 7a). A2E treatment also did not alter GSSG content when the cells were kept in the dark. However, combining A2E treatment and subsequent blue light exposure (400, 440, and 480 nm) produced a significant increase in GSSG content (Fig. 7a). In contrast, red light (630 nm) less affected the GSSG content of A2E-loaded RPE cells. These results were confirmed by the observed decreases in the GSH/GSSG ratio in the blue range (Fig. 7c). Total glutathione levels measured under the same conditions, were higher at 400 and 480 nm, whereas they remained near A2E-treated dark control level at 440 nm and 630 nm (Fig. 7b). This increase in total glutathione levels, restricted to 400 and 480 nm, indicates that GSH oxidation was compensated by an increase in glutathione synthesis at these wavelengths. Such glutathione synthesis was not observed at 440 nm, suggesting that this condition represents a more highly toxic condition producing oxidative stress that impairs glutathione synthesis.

## Discussion

### Oxidative stress signature in AMD

Ageing has been modeled in many studies by loading RPE cells with A2E, a prominent component of lipofuscin. This model enabled investigators to demonstrate that blue light exposure generates A2E photosensitization^6,28^, leading to cell death^8,10,12,19–25,29–32^. However, no study has precisely defined the wavelengths which induce the greatest cell damage, as these studies used either white light^33,34^ or broad bands of blue light, ranging from 390 to 550 nm^8^. Furthermore, these light were not filtered according to the eye optics in order to have them normalized to the daylight received onto the retina. In a recent study, we have quantified the apoptosis of A2E-loaded RPE cells induced by these narrow 10 nm-wide wavelength bands after normalizing light exposure to sun light reaching the retina in vivo^13^. Here we have further quantified the production of the two main ROS, hydrogen peroxide and superoxide anions, in the same A2E-loaded RPE cell model. Our results show that hydrogen peroxide is already produced in control RPE cells, without A2E, by blue illumination with the peak at the blue 395–405 nm band. Hydrogen peroxide levels were significantly higher (10-fold) in A2E-loaded RPE cells following illumination within the 415–445 nm range. The 1.5-fold increase in superoxide anion was less important, but still significant, with a plateau from 415 to 475 nm. Of note, the superoxide anion is a metabolic intermediate that is rapidly transformed into the more stable hydrogen peroxide by a dismutation reaction. This transformation could explain the observed higher hydrogen peroxide concentrations. Here we demonstrate that the peak spectral range for hydrogen peroxide synthesis is included in the peak of phototoxicity, which is itself within the range of superoxide anion synthesis.

### Mitochondrial changes under oxidative stress in RPE cells

A major source of intracellular ROS is the mitochondrial electron transport chain^35,36^. Furthermore, molecules of the respiratory chain, such as flavins and cytochrome oxidases, can directly absorb blue–violet light thus generating an increase of ROS and oxidative stress in cells^37–40^. A2E itself can inhibit mitochondrial ATP synthesis in RPE cells^41^. The cellular stress response has been shown in some other mammalian cell types exposed to hypoxia or viral infection^42–44^ by the modified distribution of mitochondria around the nucleus, which can thus play an important role on gene transcription^44^. Similarly, such perinuclear mitochondrial redistribution with morphological changes has been reported for ARPE-19 cells following exposure to blue near-UV light (400 nm), but not to other blue wavelengths (420 and 435 nm)^45^. Light at 405 nm was also reported to generate elongated and giant mitochondria with a decrease in their membrane potential and energy production in the same cell line^46^. We observed similar mitochondrial redistribution in A2E-loaded RPE cells following blue-light illumination (440 nm), suggesting that mitochondria are recruited at the nuclei to elicit a metabolic response with potential changes in gene transcription. However, in the previous study^45^, the redistribution of mitochondria around the nucleus was only observed in ARPE-19 cells without A2E at 400 nm but not at 420 and 435 nm. Their conditions highly differ to our experimental conditions at 400 nm because we used light irradiances normalized to the solar light reaching the retina (Fig. 1a). As a consequence, we used an eightfold lower irradiance at 400 nm (0.11 mW/cm^2^ for 15 h, 6 J/cm^2^) than the irradiance used in the study on ARPE-19 cells for the same wavelength (1.55 mW/cm^2^ for 9 h, 50 J/cm^2^). The difference in irradiance levels explains the different results observed at 400 nm. At 440 nm, our irradiance levels (1.09 mW/cm^2^ for 15 h, 58 J/cm^2^) are comparable with those applied in this previous study at 435 nm (1.35 mW/cm^2^ for 9 h, 44J/cm^2^). In the condition without addition of A2E, we did not see any morphological change as observed in the study done on ARPE-19 cells with no A2E. By contrast, when adding A2E, which photosensitized at 440 nm, we observed the mitochondrial redistribution around the nucleus. In conclusion, both our and their studies demonstrated that this redistribution of mitochondria around the nucleus is observed upon toxic photosensitizing conditions. We also found that the change in mitochondrial distribution was correlated with decreased oxidative phosphorylation due to Complex I impairment. Therefore, this change in mitochondrial physiology may compensate for the decreased efficiency of ATP production and, together with their redistribution around the nucleus, to restrict the metabolic supply for nuclear gene expression. Finally, we also observed a decrease in cell membrane potential in A2E-loaded cells exposed to blue light, as also reported for ARPE-19 cells. However, we further defined the spectral range that produces decreased mitochondrial membrane potential finding a plateau from 405 to 455 nm. This spectral range is consistent with those generating RPE apoptosis^13^ and the maximum hydrogen peroxide synthesis (see above). Thus, this mitochondrial dysfunction is likely to play an important role in the induction of RPE cell apoptosis due to hydrogen peroxide synthesis.

### Antioxidant mechanisms in A2E-loaded RPE cells

The main antioxidant enzymes are superoxide dismutases (SOD) and catalase; SOD catalyze the transformation of superoxide anions into hydrogen peroxide, whereas catalase reduces hydrogen peroxide into water and oxygen. These enzymatic mechanisms were reported to be suppressed by light in lipofuscin-loaded RPE cells^47^. These enzymatic mechanisms are complemented by a non-enzymatic antioxidant system, glutathione. In this study, we observed that the mRNA levels of antioxidant enzyme SOD2, catalase, and glutathione peroxidase 1, decreased in A2E-loaded cells exposed to blue light. Surprisingly, the level of catalase mRNA was already highly suppressed by A2E, which could explain the surge in hydrogen peroxide generated in A2E-loaded cells, even in darkness. However, the levels of catalase activity were not consistent with those of its mRNA, suggesting that protein and mRNA levels were not directly correlated for this enzyme. Similarly, the mRNA levels of SOD explain neither the reduced SOD activity in A2E-loaded cells nor its increase in SOD activity when they were exposed to blue light. Indeed, SOD activity decreased by 50% upon A2E treatment of cells which were then kept in darkness, resulting in an increase in superoxide anion levels due to its reduced transformation into hydrogen peroxide. The mRNA transcription of antioxidant proteins like SOD was downregulated by the high toxicity of blue light by contrast to the protein activities. This result is consistent with the delay in mRNA translation with respect to mRNA transcription. For catalase, the greater light-induced reduction in protein activity suggest that light itself could play a role in the inactivation or even degradation of the protein as described in plants^48^. Concerning the reduction in SOD activity in the presence of A2E, its functional recovery upon light exposure in A2E-loaded RPE cells suggest that A2E could be an inhibitor of the enzyme activity as some other mitochondrial enzymes^49^, but that this binding or inhibition can be modulated or inactivated by light. The oxidative stress-induced by blue light was also suggested by an increase in oxidized glutathione levels in A2E-loaded cells. Total glutathione levels (oxidized + reduced) increased following exposure to the less toxic portions of the blue range (400 and 480 nm), but less under the most toxic wavelength (440 nm). This suggests that, RPE cells can actively neutralize ROS under oxidative conditions by generating glutathione only if the oxidative stress remains below a certain oxidative threshold.

### Retinal diseases and therapeutic strategies: light filtering, mitochondrial targeting, biomarkers

Oxidative stress is thought to play a major role in the progression of AMD^50–52^. Blue light, which is now considered to be a risk factor for the development of AMD, can significantly contribute to oxidative stress and the pathogenesis of AMD^26,53^. This blue light toxicity is related to the age-dependent accumulation of lipofuscin and its photosensitizing constituent A2E. As a consequence, many groups have examined the molecular mechanisms underlying blue-light toxicity using AMD cell models, often relying on lipofuscin or A2E-loaded RPE cells^8,10,12,19–25,29–32^. We have recently reported the most toxic wavelengths in A2E-loaded RPE cells by measuring light-induced apoptosis^13^. Here we have confirmed the higher toxicity of the 415–455 nm spectral band by analyzing the different induced molecular mechanisms of ROS production and mitochondrial impairment. Blue photosensitization of A2E is producing ROS leading to mitochondrial damage and ultimately cell death. The large alteration of mitochondrial function suggests that this organelle may be a potential target for the prevention of AMD, or even treatment of the disease. Sheu et al.^54^ have proposed the stimulation of mitochondrial function with Resveratrol to protect RPE cells from oxidative damage. In preventive care, broadband blue-light filtering has been proposed in eye glasses and even intraocular lenses during cataract surgery^33,55,56^. Our study supports precise filtering of the most toxic wavelengths in the blue–violet range. This selectivity should limit the alteration of color vision and chrono-biological regulations controlled by intrinsically sensitive retinal ganglion cells (480 nm). This study should therefore help define the best filtering devices to prevent AMD and its progression.

## Material and methods

### Cell model

Retinal pigment epithelium cells (RPE cells) were extracted from porcine eyes as previously described^13^. Primary RPE cells from 20 eyes were plated in 60 mm Petri dishes in Dulbecco’s Modified Eagle Medium (DMEM, Life Technologies, Carlsbad, CA, USA), 20% Fetal Bovine Serum (Eurobio, Courtaboeuf, France) and 10 mg/mL gentamycin (Life Technologies) and allowed to grow to confluence in a controlled atmosphere under 5% CO_2_ at 37 °C. Culture medium was renewed 24 h after first seeding. Upon reaching confluence, cells were detached by incubation in 0.05% trypsin-EDTA (Life Technologies) for 5 min at 37 °C, resuspended in culture medium, counted, and seeded at 75,000 cells per well in black, clear bottom, 96-well plates (Corning, NY, USA). For immunostaining experiments black 96-well plates were purchase from IBIDI (Munich, Germany). For oximetry and superoxide dismutase activity experiments, cells were seeded in black clear bottom 6-well plate (Iwaki, Chiba, Japan) at 1,400,000 cells per well. From this step on, all experiments were conducted in darkness under moderate red light. Three days after seeding, confluent cells were treated for 6 h with 0, 10, 12.5, or 20 µM A2E (Orga-Link, Magny-les-Hameaux, France) in DMEM without serum for all conditions. The absence of serum was required to remove any light absorbing molecule in the culture medium and also to prevent fast cell division while not affecting cell viability. Moderate concentrations of A2E (10 and 12.5 µM) were used for immunostaining and oximetry experiments to limit light toxicity and facilitate measurements. DMSO (Sigma-Aldrich, St Louis, MO, USA) was adjusted to a final concentration of 0.1% for all conditions. After A2E treatment, cells were washed twice with modified DMEM (medium without any photosensitizer, such as phenol red, riboflavin, folic acid, or aromatic amino acids, Life Technologies) and exposed to light for 15 h.

### Light conditions

Cells were exposed to 10 nm-wide spectral bands produced by a purpose-made LED-based fibered light device for 15 h as previously described^13^. Central wavelengths of the narrow light bands were equally distributed from 390 to 520 nm in 10 nm increments (14 narrow bands available). A 15th band with a central wavelength set at 630 nm was added. To mimic physiological light conditions on the retina, RPE cells were exposed to a normalized light spectrum obtained by applying the ocular media filtering onto the referenced solar spectrum (ASTM G173-03, International standard ISO 9845-1, 1992); blue light is partly filtered by the anterior ocular media as a natural protector. The maximum irradiance level was obtained for the light band centered at 630 nm, fixed at 1.5 mW/cm^2^. Irradiance level, spectral, and uniformity measurements were assessed using the calibrated spectroradiometer JAZ (Ocean Optics, Dunedin, USA). Cells were directly characterized after light exposure.

### Quantification of intracellular ROS

After 15 h of light exposure, two ROS levels were quantified in RPE cells. The level of hydrogen peroxide was assessed using the ROS-Glo H_2_O_2_ Assay kit (Promega, Madison, WI, USA) according to the manufacturer’s protocol. Briefly, cells were incubated with H_2_O_2_ Substrate Solution for 2 h before the end of exposure. Then, ROS-GLO Detection Solution was added at the end of light exposure and the cells incubated for another 20 min before luminescence reading on an Infinite M1000 microplate reader (Tecan, Männedorf, Switzerland). Superoxide anion levels were quantified using the MitoSOX Red Mitochondrial Superoxide Indicator kit (Life Technologies). MitoSOX reagent working solution (5 µM, 0.1% DMSO) was prepared by diluting MitoSOX reagent stock solution (5 mM in DMSO) with modified DMEM and the solution added to cells at the beginning of light exposure. Fluorescence was quantified at ex 510/em 580 nm on a microplate reader (Infinite M1000, Tecan).

### ATP content

After light exposure and after a rest period of 6 h, CellTiter-Glo Reagent (Promega) was added to cell culture medium to quantify ATP content according to the manufacturer’s protocol. Well plates were shaken during 15 min on an orbital shaker to induce cell lysis and then incubated at room temperature for 10 min before luminescence reading on a microplate reader (Infinite M1000, Tecan), see Supplementary Information 1 for results.

### Immunocytochemistry and image analysis

After light exposure, cells were washed twice with PBS (Life Technologies) and fixed with 4% formaldehyde (Merck-Millipore, Bellerica, MA, USA) for 15 min at room temperature. Cells were then washed with PBS and permeabilization was performed with PBS-Triton (0.1%) for 5 min at room temperature. After three washes of 5 min in PBS, non-specific antibody binding sites were saturated for 1 h at room temperature in PBS-1% BSA-0.05% Tween-20. ATP synthase (A21351, Life Technologies) and tight junctions (ZO-1, 61-7300, Life technologies) were stained by overnight incubation at 4 °C with primary antibodies diluted in saturation buffer. Secondary goat anti-mouse IgG and goat anti-rabbit IgG conjugated to either Alexa Fluor 594 or Alexa Fluor 488 (A11005 and A11008, Life Technologies) were applied for 2 h at room temperature. Nuclei were counter-stained with 4′,6-diamidino-2-phenylindole (DAPI). Imaging was performed on an inverted confocal microscope (FV-1200 equipped with GaAsP detectors, Olympus, Tokyo, Japan) using identical settings for each A2E and light conditions (60× oil objective was used for image quantification and 60× oil objective plus four times zoom was used for illustrations). All reagents were purchased from Sigma-Aldrich unless otherwise specified. ‘Cell’ module of Imaris software (Bitplane, Zurich, Switzerland) was used to analyze subcellular localization of mitochondria on 3D images. Cell area, cell cytoplasm number of mitochondria and cell mitochondria fluorescence intensity were evaluated.

### Oxigraphy

After light exposure, cells were washed twice with PBS (Life Technologies), incubated 5 min at 37 °C with 0.25% trypsin without EDTA (Life Technologies) and detached from the 6-well plates. Trypsinization was stopped by adding culture medium to the cell suspension. After centrifugation at 800 r.p.m. at 37 °C for 5 min, pellets were resuspended in a buffer containing 0.5 mM EGTA, 3 mM MgCl_2_.6H_2_O, 60 mM k-lactobionate, 20 mM taurine, 10 mM KH_2_PO_4_, 20 mM HEPES, 110 mM sucrose, and 1 mg/mL BSA at pH = 7.1, and placed in the chamber of an oxygraph (Oroboros Instruments, Innsbruck, Austria) to measure O_2_ consumption. To permeabilize the plasma membrane, digitonin (15 µg/1 × 10^6^ cells) was added to the cell suspension. Malate (5 mM) and pyruvate (5 mM) were then added to provide NADH to Complex I and define the respiration rate driven by Complex I. Activation of ATP synthesis was induced by the addition of 1.5 mM ADP. Thus, Complex I-driven respiration coupled to ATP synthesis was obtained. Next, glutamate (5 mM) was added to provide another source of NADH. The addition of succinate (10 mM) allowed reconstitution of the Krebs cycle function with the activation of the succinate dehydrogenase. Thus, we determined maximal respiration of the mitochondrial chain induced by the competition of respiration driven by Complex I and Complex II. The addition of rotenone (10 µM) inhibited electron transfer from Complex I to coenzyme Q and allowed the measurement of Complex II-driven respiration. Oligomycin supplementation (8 µg/mL) inhibited ATP synthesis and the uncoupled respiration driven by Complex II was determined. Finally, carbonyl cyanide-4-(trifluoromethoxy)phenylhydrazone (FCCP, 1 µM) was added to verify the quality of the plasma membrane permeabilization by digitonin. All reagents were purchased from Sigma-Aldrich, unless otherwise specified.

### Mitochondrial membrane potential

Mitochondrial membrane potential was measured using the Mito-ID membrane potential cytotoxicity kit (Enzo Life Sciences, Farmingdale, NY, USA). Carbonyl cyanide 3-chlorophenylhydrazone (CCCP, 4 µM) was added 30 min before the end of light exposure to a few wells to abolish the mitochondrial membrane potential as a positive control. At the end of light exposure, the dye was directly dispensed into each well and the plate incubated for 30 min at room temperature. Dye fluorescence was quantified on a microplate reader at ex 490 nm/em 590 nm (Infinite M1000, Tecan) and images for illustration were acquired with an automated microscope equipped with a ×20 Objective (Arrayscan, Thermo Scientific).

### Real-time PCR

Total RNA was isolated using the RNeasy Micro Kit (Qiagen, Hilden, Germany). RNA quality and quantity were assessed by spectrophotometry (NanoDrop, Thermo Scientific). Synthesis of cDNA was performed using SuperScript II (Life Technologies) with random primers (Promega), 10 mM dNTP (Life Technologies) and 0.1 M DTT (Life Technologies). The mix was incubated 10 min at room temperature then 50 min at 42 °C, and finally 15 min at 70 °C. Real-time PCR was performed using a StepOne device (Life Technologies) with SYBR Green (Life Technologies) and catalase forward (5′-CTCGTGGGTTTGCAGTGAAA-3′) and reverse primers (5′-GAGACTCAGGACGTAGGCTC-3′), SOD1 forward (5′-AGTGCAGGTCCTCACTTCAA-3′) and reverse primers (5′-CATTTCCACCTCTGCCCAAG-3′), SOD2 forward (5′-TGAACAACCTGAACGTCGTG-3′) and reverse primers (5′-AGCGGTCAACTTCTCCTTGA-3′) and GPX1 forward (5′-ATTGCCTCAAGTACGTCCGA-3′) and reverse primers (5′-CATTGCGACACACTGGAGAC-3′). The data were normalized to 18S RNA which was simultaneously amplified using 18S forward (5′-AGTCGGCATCGTTTATGGTC-3′) and reverse primers (5′-CGCGGTTCTATTTTGTTGGT-3′). Primers were purchased from Sigma-Aldrich. RT-PCR amplification products were purified and sequenced using the Sanger method. To validate the specificity of the RT-PCR amplifications, sequences were compared to the pig genome.

### SOD activity

Superoxide dismutase activity was measured using the Superoxide Dismutase Assay Kit (Cayman, Ann Arbor, MI, USA) on protein extracts. After 15 h of light exposure, cells were collected in cold buffer (20 mM HEPES, pH 7.2, 1 mM EGTA, 210 mM mannitol, 70 mM sucrose, all from Sigma-Aldrich), sonicated and centrifuged to separate mitochondrial and cytoplasmic proteins according to the manufacturer’s protocol. SOD activity in the samples was quantified using a standard curve obtained with bovine erythrocyte SOD supplied in the kit. Absorbance was measured at 450 nm using a microplate reader (Infinite M1000, Tecan).

### Catalase activity

After 15 h of light exposure, cells were washed in ice-cold phosphate-buffered saline (PBS, Life Technologies), lysed on ice in Cell Lysis Buffer (Cell Signaling Technology, Boston, MA, USA) supplemented with protease inhibitor cocktail (Roche, Basel, Switzerland), and sonicated. Cell lysates were centrifuged, supernatants collected and catalase activity was assessed using the Amplex Red Catalase Assay Kit (Life Technologies) on cell lysates, according to the manufacturer’s protocol. Fluorescence was measured at ex 540 nm/em 590 nm using a microplate reader (Infinite M1000, Tecan).

### Measurement of the GSH/GSSG ratio

The ratio of reduced and oxidized forms of glutathione was measured using the GSH/GSSG-Glo Assay kit (Promega) according to the manufacturer’s protocol at the end of light exposure. Cells were treated either with Total or Oxidized Glutathione Reagent for 5 min under shaking. Luciferin Generation Reagent was then added to all wells and the plates incubated 30 min before adding Luciferin Detection Reagent. Luminescence was read on a microplate reader (Infinite M1000, Tecan) and the GSH/GSSG ratio was determined.

### Statistical analysis

All experiments were repeated at least three times, results for an assay were therefore the averaged values of at least three independent experiments. Each individual experiment was run in technical triplicate, meaning that each condition of that experiment was the averaged value from three wells on the well plates except for catalase and SOD activities (technical duplicate), oxygraphy and mitochondrial distribution analysis (one well per experiment) due to measurement complexity. The data were represented as mean ± SEM. Statistical analyzes were performed using Statistica software (StatSoft, Tulsa, OK, USA). Two-way ANOVA with repeated measures followed by Dunnett’s post hoc tests were used to compare variances of all groups (at each A2E concentration and each light condition) to the dark control groups. Differences between samples and dark controls were considered to be significant when *p* < 0.05 (▲/*), *p* < 0.01 (▲▲/**), or *p* < 0.001 (▲▲▲/***). Triangles (▲) refer to untreated and stars (*) to A2E-treated dark controls.

## Electronic supplementary material

Supplementary information 1

Supplementary information 2
