# Supplementary figures and images for: Light action spectrum on oxidative stress and mitochondrial damage in A2E-loaded retinal pigment epithelium cells
Source: Cell Death Dis. 2018 Feb 19;9(3):287. doi: 10.1038/s41419-018-0331-5 (PMC5833722; doi:10.1038/s41419-018-0331-5)

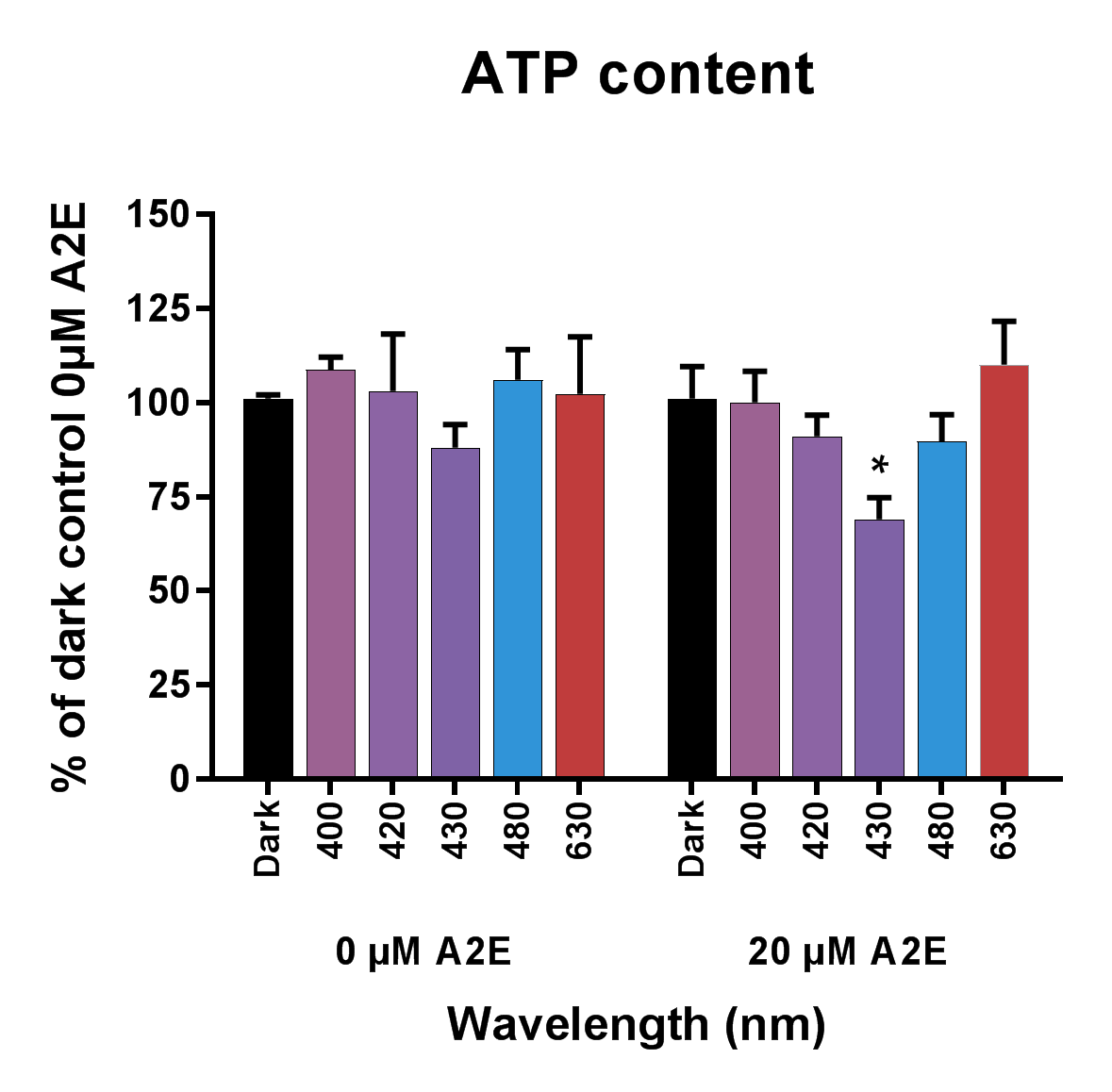

Supplement: Supplementary file 1 — Supplementary information 1 [file 41419_2018_331_MOESM1_ESM.tif]
