# Supplementary material for: Light action spectrum on oxidative stress and mitochondrial damage in A2E-loaded retinal pigment epithelium cells
Source: Cell Death Dis. 2018 Feb 19;9(3):287. doi: 10.1038/s41419-018-0331-5 (PMC5833722; doi:10.1038/s41419-018-0331-5)
Supplement: Supplementary file 2 — Supplementary information 2 [file 41419_2018_331_MOESM2_ESM.docx]

**Supplementary information 1:** ATP content was measured after light exposure and a rest period of 6 h, CellTiter-Glo^®^ Reagent (Promega) was added to cell culture medium according to the manufacturer’s protocol and luminescence was read on a microplate reader (Tecan). Significant difference with treated control was represented by stars p<0.05 (*).
